# Unveiling sex-disparities and the impact of gender-affirming hormone therapy on periodontal health

**DOI:** 10.3389/fdmed.2024.1430193

**Published:** 2024-09-16

**Authors:** Cristina Cunha Villar, Mariane Cristina Sloniak, Josiane Betim de Assis, Renata Cassiano Porto, Giuseppe Alexandre Romito

**Affiliations:** ^1^ Discipline of Periodontics, Department of Stomatology, University of São Paulo School of Dentistry, São Paulo, Brazil; ^2^ Department of Immunology, Institute of Biomedical Science, University of São Paulo, São Paulo, Brazil

**Keywords:** periodontitis, immunity, gonadal steroid hormones, biological sex, gender-affirming care

## Abstract

**Introduction:**

As personalized medicine advances, the need to explore periodontal health across different sexes and gender identities becomes crucial. This narrative review addresses the gap in understanding how biological sex and gender-affirming hormone therapy (GAHT) influence periodontitis risk.

**Results:**

Research has uncovered significant sex-based immunological disparities driven by X and Y chromosome gene expression and sex-hormones, which may influence susceptibility to periodontitis. Additionally, preliminary findings suggest that GAHT, particularly testosterone therapy in transgender men, could exacerbate pro-inflammatory cytokine production and alter immune cell responses, which may exacerbate inflammatory pathways crucial in the progression of periodontitis. Conversely, the effects of estrogen therapy in transgender women, although less extensively studied, suggest modifications in B cell functionality. These observations highlight the complex role of GAHT in modulating immune responses that are central to the development and exacerbation of periodontal disease.

**Discussion:**

The review highlights a complex interaction between sex hormones, gene expression patterns, immune responses, and periodontitis risk. While cisgender males show increased susceptibility to periodontitis that could be linked to specific immune pathways, GAHT appears to modify these pathways in transgender individuals, potentially altering their risk and disease progression patterns.

**Conclusion:**

There is a critical need for more focused research on the direct impacts of GAHT on periodontal health. Understanding the nuances of immune modulation by GAHT will aid in crafting personalized periodontal care for transgender individuals, aligning with the broader goals of inclusive and effective healthcare.

## Introduction

In the evolving paradigm of personalized medicine, it is imperative to address inclusivity across all genders and sexes in biomedical research. This approach is not merely a matter of ethical consideration but a foundational pillar for achieving equitable and effective healthcare outcomes. The importance of this inclusive perspective is notably reflected in the context of periodontitis, a leading cause of tooth loss worldwide, and a significant public health concern with substantial implications for individuals' quality of life and overall well-being (1). Periodontitis, characterized as a multifactorial chronic inflammatory condition, results from the complex and dynamic host-microbiota-exposome interplay (2–4). These factors collectively shape a highly individualized immune response, contributing to the patient disease susceptibility (2–4).

Recent epidemiological studies have unveiled sex-based disparities in the prevalence of periodontitis, with cisgender males being 11.95% more likely to develop the condition compared to cis females (5). This discrepancy underscores the complex, sex-specific regulatory mechanisms governing the immune system, which are influenced by the interplay of sex hormonal and chromosomal factors (6). The X chromosome plays a pivotal role in this regulatory divergence, hosting an array of immune-related genes such as the Toll-like receptor (TLR)-7, CD40, CXCR3, and the transcription factor FOXP3 (7–9). In cisgender females, the presence of two X chromosomes adds complexity to immune system regulation due to X-chromosome inactivation. This mechanism balances gene expression by turning off one X chromosome in each cell. Despite this, approximately 15% of X-linked genes escape silencing, resulting in a distinct biallelic expression pattern (7). In contrast, cisgender males, with only one X chromosome, are more susceptible to mutations in X-linked genes (6, 10, 11). Sex hormones add additional complexity to immune regulation by interacting with receptors on both innate and adaptive immune cells, thereby influencing immune responses (6, 12). The impact of these interactions is highly nuanced, varying with the nature of the immune stimulus, the target cell, and sex-hormone concentrations (12).

The complexity of this landscape is further increased when considering the transgender population undergoing gender-affirming hormone therapy (GAHT) (13). GAHT plays an essential role in alleviating gender dysphoria and improving psychological well-being by introducing exogenous hormones. This intervention likely alters the immune system’s regulatory mechanisms, potentially affecting susceptibility to autoimmune and inflammatory diseases. Despite its significance, the impacts of GAHT on immunological responses and vulnerability to infectious and inflammatory conditions remain largely underexplored. Firstly, while sex-based differences in immune function are well-documented, it is unclear how these differences translate to transgender individuals undergoing hormone therapy. The interplay between sex hormones such as estrogen and testosterone and immune function is complex and not fully understood. Furthermore, transgender individuals are significantly underrepresented in scientific research, leading to a lack of long-term studies focusing on the immunological impacts of hormone therapy in this population. This gap in research is particularly concerning given the increasing number of transgender adolescents and adults seeking gender-affirming care, highlighting the urgent need for more comprehensive studies to ensure their positive health outcomes (14, 15). In a landscape that increasingly values gender diversity and personalized healthcare, it becomes crucial to understand how biological sex and GAHT impact periodontal health. This review aims to bridge this knowledge gap by exploring how biological sex and GAHT modify immune responses, thereby influencing the risk of periodontitis.

### Immune responses in periodontal disease

Periodontitis, recognized as a chronic inflammatory disease, arises from a complex interplay between microbial dysbiosis, environmental factors, and the host’s immune response. This complex interplay transcends simple microbial pathogenicity, revealing a scenario where an altered inflammatory state drives cycles of microbial dysbiosis and tissue destruction (2–4, 16).

Periodontitis is marked by increased activity of various immune cells, creating a harmful inflammatory environment. Neutrophils, for instance, play a dual role. While essential for controlling bacterial infections, neutrophils become hiperactive in periodontitis. This overactivity results in excessive production of enzymes and pro-inflammatory mediators that exacerbate tissue damage (3, 17, 18). Similarly, macrophages in periodontitis are often induced towards a pro-inflammatory M1 phenotype, secreting large amounts of cytokines such as interferon-gamma (IFN-*γ*), tumor necrosis factor-alpha (TNF-α), and interleukins (IL) (e.g., IL-6, IL-12), along with matrix metalloproteinases (MMPs), further intensifying the inflammation (19).

The pathogenesis of periodontitis is also critically influenced by the orchestrated interplay among CD4^+^ T cell subsets, including Th1, Th2, Th17, and regulatory T (Treg) cells (20). The dynamic equilibrium between the pro-inflammatory effects of Th17 cells and the immunosuppressive actions of Treg cells is crucial for maintaining periodontal health while its imbalance drives disease progression (21). Th17 cells, found in increased numbers in periodontitis, promote inflammation and tissue destruction by producing IL-17 (21, 22). In contrast, Treg cells counteract these effects by releasing anti-inflammatory cytokines like IL-10 and transforming growth factor-beta (TGF-β), thus mitigating Th17-driven damage (23). Th1 cells, known for their IFN-γ production, are key in driving the cellular immune response, including activating neutrophils and macrophages (24). Although essential for defending against pathogens, this response can lead to increased periodontal tissue damage if it becomes dysregulated. Conversely, Th2 cells contribute to the humoral immune response by producing cytokines such as IL-4 and IL-5, which help in antibody production. Depending on the disease stage and individual immunological context, this can either help control or worsen periodontal damage (24).

B cells play multifaceted roles including antibody production, cytokine secretion, antigen presentation, and maintaining immunological memory (25). In healthy states, memory B cells are sparse near the apical region of the junctional epithelium (18, 26), strategically positioned to defend against constant microbial invasions. However, in periodontitis, this balance is significantly disrupted, marked by a notable increase in B cells and plasma cells, particularly those producing IgG and IgM (27). A shift towards a predominance of antibody-secreting cells over memory B cells indicates a heightened immune reaction (28), with a significant role in the development and progression of periodontitis. Research involving both humans and animals underscores the contribution of B cells to periodontal tissue destruction. Through the release of pro-inflammatory cytokines such as IL-1β and TNF-α, along with the expression of RANKL, B cells exacerbate periodontal damage (25, 29–32).

### Differences in immune responses and periodontal health in cisgender populations

#### Influence of biological sex on immune responses

Both sex hormones and genetic factors significantly shape immune functions, influencing disease susceptibility and treatment responses across sexes. Typically, females exhibit greater immune reactivity than males, a trait influenced by the X chromosome. The phenomenon of X chromosome inactivation gives women a biological advantage, allowing for heterogeneous expression of gene mutations. This heterogeneity often results in females either not developing X-linked diseases or experiencing them in a milder form compared to males who possess the same genetic variant (33, 34). The X chromosome encodes several proteins that contribute to sex-based differences in immune responses, including Toll-Like Receptors (e.g., TLR7, TLR8), cytokine receptors (e.g., IL2Rg, IL9R), and transcription factors such as FOXP3 and NKRF (35). Additionally, approximately 10% of the genomic non-coding microRNAs (miRNAs) reside on the X chromosome. Specific miRNAs, such as miR-223, regulate neutrophil differentiation and suppress inflammatory responses, whereas others like miR-221/222 modulate STAT3 expression. A group including miR-20a/b, miR-106 a/b, miR-424, and miR-513 influences the stability and translation of the PD-L1 transcript, which inhibits activated immune cells (36, 37).

Research using a mouse model of multiple sclerosis has shown that polymorphisms in genes on the Y chromosome can increase susceptibility to experimental encephalomyelitis, supporting the notion that sex-based genetic factors are linked to autoimmune diseases (38, 39). Further studies have indicated that genetic variations on the Y chromosome not only heighten susceptibility to influenza A virus infection in male mice but also affect their survival post-infection, a phenomenon associated with an increase in IL-17-producing γδ T cells (40). Moreover, preclinical research on Coxsackievirus B3, linked with myocarditis and higher mortality in individuals under 40, suggests that reduced survival rates in infected male mice could be due to epigenetic changes driven by polymorphisms on the Y chromosome’s heterochromatic regions (41).

The biological differences in sex steroid concentrations significantly influence the immune response regulation. Steroid hormones are produced mainly in the adrenal cortex, gonads and placenta. During steroidogenesis, mitochondrial metabolism of cholesterol originates pregnenolone, the precursor of all steroid hormones (42). Estrogens, specifically estrone (E1), estradiol (E2), and estriol (E3), along with their receptors (ER) found on several immune cells, regulate not only secondary sexual characteristics but also modulate various immune processes (43, 44). Estrogen therapy, including ERβ agonists, promotes IL-4-induced M2 gene expression in asthma models (45) and reduces the severity and mortality of experimental autoimmune encephalomyelitis (EAE) through its effects on T cells, demonstrating a profound impact across both innate and adaptive immunity (46). Estradiol and progesterone have been shown to delay neutrophil apoptosis, thereby prolonging neutrophil function in healthy adults (47), this may explain the observed decline in blood neutrophils in postmenopausal women. Additionally, estrogen’s interaction with peripheral tolerance mechanisms suggests a role in modulating T cell differentiation, particularly influencing the differentiation of CD4+ T cells into Treg cells (48). The ability of estradiol to boost the humoral immune response, increasing immunoglobulin synthesis in a concentration-dependent manner, further highlights its critical role in immune modulation (49). Studies in Rhesus macaques have confirmed that estrogen significantly increases the frequency of antibody-secreting cells, with variations that align with the menstrual cycle (50).

Like estrogen, progesterone serves roles beyond its well-known functions in female sexual and reproductive health. Research conducted *in vitro* has demonstrated that progesterone exerts an immunosuppressive effect. It inhibits the production of inflammatory cytokines such as TNF-α, IL-1β, IL-6, and IL-12 as well as the expression of costimulatory molecules such as CD40, CD80, and CD86 by dendritic cells activated through TLR3 and TLR4 ligands (51, 52). Synthetic derivatives of progesterone, known as progestins, have also been shown to decrease the release of IL-6, IL-8, and MCP-1 by endometrial stromal cells (53). Further studies revealed that both progesterone and dexamethasone can induce apoptosis of mouse CD4^+^ T cells, while Treg cells are resistant to these effects (54). Notably, progesterone also enhances the differentiation of naïve T cells into Treg cells while reducing the production of pro-inflammatory Th17 cells (55). Additionally, it shifts the immune response towards Th2 dominance, diminishing Th1 responses *in vitro* (56).

Testosterone, the primary male hormone, interacts with cells manly via the androgen receptor (AR). While AR plays a critical role in neutrophil development (57), testosterone administration has been observed to decrease the microbicidal activity of these cells (58). Interestingly, female natural killer cells produce more IFN-γ than their male counterparts, an effect that can be reduced by testosterone exposure (59, 60). Additionally, androgens influence circulating B cells and serum antibody levels, impacting B cell tolerance (61). Furthermore, males have been shown to exhibit higher thymic expression of the autoimmune regulatory gene (AIRE) compared to females, a difference regulated by androgens and their receptors, which significantly shapes central T cell tolerance (62). It is worth noting, however, that the biological activity of testosterone can be peripherally altered. Testosterone can be naturally converted into dihydrotestosterone (DHT), which acts via AR to amplify its effects; inactivated through its conversion to androstenedione; or converted to estradiol during ovarian steroidogenesis and peripheral modulation via aromatase. The estradiol produced from testosterone diversifies the hormone’s activity by acting through ER, further influencing physiological processes (42, 63, 64).

In sum, while estrogens significantly boost the immune response in cisgender women, leading to more effective pathogen clearance and a stronger humoral response, progesterone’s role is more complex, appearing to be crucial in balancing immune functions, which is critical for maintaining tolerance during pregnancy. On the other hand, increased androgen activity, particularly testosterone, in cisgender men tends to foster anti-inflammatory responses across both innate and adaptive immune systems. Thus, the impact of sexual dimorphism on immune responses is highly significant and is likely to explain disparities in susceptibility to autoimmune and infectious diseases between sexes.

#### Implications for periodontal disease

Sex-based immune differences are likely to significantly impact periodontal disease outcomes in cisgender populations. Enhanced expression of TLR4 in male neutrophils correlates with increased TNF-α production, either constitutively or following lipopolysaccharide (LPS) stimulation (65). Similarly, male peripheral blood mononuclear cells (PBMCs) also produce more TNF-α when exposed to LPS (66), indicating a heightened pro-inflammatory response of male inate immune cells to microbial challenges. Conversely, testosterone treatment *in vitro* reduces TLR4 expression and TNF-α production in macrophages, suggesting an anti-inflammatory effect (67–69). The balance in the defense system in periodontal disease is fundamental, for example, while controlled TNF-α production assists in clearing periodontopathogens, their unregulated production can lead to the destruction of periodontal tissues (2).

In females, elevated phagocytic activity (70) and antigen presentation efficiency (71) by antigen-presenting cells (APCs) enhance the recognition and elimination of pathogens to limit tissue damage. Estrogen and progesterone direct the immune response towards Th2 and Treg dominance, boosting humoral responses and suppressing Th17 cell proliferation and IL-17 production (55, 72), thereby potentially protecting against periodontal destruction by moderating inflammation.

The literature presents mixed findings on the hormonal influence on periodontal disease. Estrogen deficiency, as observed in ovariectomy models, generally exacerbates alveolar bone loss in experimental periodontitis (73–75). Ovariectomized rats exhibit greater osteoclast activity and bone loss following ligature-induced periodontitis (74), and similar effects are seen in sheep, with increased alveolar bone loss and higher IL-6 levels in diseased sites one year post-ovariectomy (75). Increased osteoclast numbers, higher RANKL expression, and decreased IFN-γ production are observed in cultures from ovariectomized mice in response to LPS (76). IFN-γ, generally produced by T cells, regulates osteoclastogenesis by interfering with the RANKL signaling pathway (77), favoring bone resorption in estrogen deficiency conditions. Ovariectomy also causes osteoporotic changes and thinning of the alveolar bone in the rat molar interradicular septum (78). Estrogen and calcitonin administration do not protect against biofilm-induced bone loss in ovariectomy models but prevent direct estrogen-deficiency-related alveolar bone loss (79). Conversely, some studies found no significant impact of ovariectomy on periodontal attachment (80) and observed reduced neutrophil and T-lymphocyte migration to the periodontal tissues following local LPS administration in ovarectomized mice (81).

Testosterone deprivation through both orchiectomy and chemical AR blockade with flutamide increases bone resorption in male rats under experimental periodontitis, though only orchiectomy also increases osteoclast counts (82). Orchiectomized animals show higher gingival IL-1β levels, while flutamide treatment decreases gingival IL-6 levels (82). In a model of ligature-induced periodontal inflammation in rats, orchiectomy has been shown to increase prostaglandin E2 (PGE2) and lipoxin A4 (LXA4) expression in gingival tissues, while IL-10 increased with subsequent testosterone supplementation (83). In this same model, AR activation through testosterone administration combined with an aromatase inhibitor induced significant increases in EGF and VEGF levels in female rats, while receptor blockade significantly increased bone loss (84). Treatment of periodontal disease further demonstrates that bone repair is significantly impaired by AR blockade, while testosterone supplementation significantly increases the inflammatory infiltrate (85). Nonetheless, long-term testosterone depletion has been shown to attenuate inflammatory bone resorption and decrease IL-1β expression in experimental periodontitis in rats (86).

Expression of specific osteoclast genes also shows sexual dimorphism, which may explain differences in bone loss between males and females (87). Additionally, variations in the microenvironment further influence osteoclast formation disparities between sexes, with males displaying higher osteoclast counts (87). Periodontal bone loss is driven and sustained by uncontrolled leukocyte infiltration, leading to an intense immunoinflammatory response. Observations indicate disparities in neutrophil recruitment, chemokine expression, and osteoclast-driven bone loss between male and female mice (87, 88). Neutrophils, the first responders at inflammatory sites, migrate via chemokine signaling and attempt to neutralize pathogens while maintaining inflammation by secreting cytokines (89). The chemokine receptors CXCR4 and CXCR2 play critical roles in these dynamics; CXCR4 retains neutrophils in the bone marrow, whereas CXCR2 facilitates their mobilization into the bloodstream (87, 90). The ligands for these receptors include CXCL12 (SDF-1) for CXCR4 and CXCL1 (KC) and CXCL2 (MIP-2) for CXCR2 (87). Notably, individuals with periodontal disease show elevated CXCL12 levels, which decrease following subgingival instrumentation (91). In a peritonitis model, female mice demonstrated lower neutrophil recruitment and a less intense inflammatory response, associated with higher CXCL12 and CXCR4 levels, compared to males (92). Additionally, studies on Ly6G + cells activated with LPS reveal that male cells express increased levels of chemokines CXCL1, CXCL2, and CXCL3, but lower levels of CXCR2, CXCR3, and CXCR4 (88). Furthermore, male neutrophils exhibit significantly higher expression of IL-1β, IL-6, and TNF-α, and over tenfold higher IL-10 and iNOS mRNA levels than females (88), suggesting a more pronounced pro-inflammatory and regulatory cytokine profile in males. Male neutrophils also exhibit a significantly elevated CXCL10 expression, more than 82 times higher when compared with females (88). This increase is critical as LPS-induced CXCL10 promotes osteoclast formation (93). Moreover, the pronounced expression of the CXCR3 and CXCL10 in periodontal disease correlates with elevated IFN-γ levels, which are instrumental in activating macrophages (87), which can contribute to the higher prevalence of periodontitis in males. Altogether, these profound implications of sexual dimorphism on immune defense mechanisms are likely to affect periodontal disease susceptibility and progression.

#### Evidence from cisgender populations

The intricate relationship between sex hormones and periodontal health varies across different life stages. While female sex hormones generally provide a protective effect against periodontal diseases, favoring Th2 and Treg responses, significant fluctuations in estrogen and progesterone during puberty, pregnancy, and menopause can intensify periodontal diseases (94, 95). These hormones influence periodontal tissues through specific receptors present in gingival fibroblasts, osteoblasts, and periodontal ligament fibroblasts (96, 97), mediating both protective and detrimental effects.

Puberty marks a critical phase where sex hormones, including testosterone, estradiol, and progesterone, surge. This hormonal increase is linked to an increase in gingival inflammation from prepubertal years to puberty (98) that exceeds what is typically observed in adults with comparable plaque levels (99). This suggests a heightened sensitivity of the periodontium to hormonal fluctuations during adolescence. This period is also characterized by shifts in the subgingival microflora, with an increase in *Prevotella intermedia* (*P. intermedia*)*, P. melaninogenica, Actinomyces odontolyticus* and *Capnocytophaga species*, which respond dynamically to hormonal changes and exacerbate gingival inflammation (100).

Further emphasizing the influence of testosterone, research across various models has revealed the dual effects of testosterone. Research using male rat models of ligature-induced periodontitis has demonstrated the effects of testosterone manipulation, employing both surgical orchiectomy (OCX) and chemical AR inhibition. Initial studies revealed that these interventions exacerbated bone loss when compared to controls with normal testosterone levels (82, 101). However, subsequent findings indicated that long-term testosterone depletion significantly reduces bone resorption, an effect reversible with exogenous testosterone supplementation (86), highlighting testosterone's pro-resorptive role in periodontal disease. This is further supported by evidence that supra-physiological testosterone levels, achieved through periodic injections, result in increased ligature-induced bone loss (101). Extending these findings to humans, data from the NHANES cohort of 755 men confirmed that elevated testosterone levels correlate with increased prevalence and severity of periodontitis (102). Additionally, studies on androgenic anabolic steroid (AAS) abuse in men have shown that AAS users not only have a higher incidence of periodontitis but also a significantly more dysbiotic microbial profile, indicating the detrimental impact of supra-physiological testosterone levels on periodontal health (103).

Pregnancy brings significant hormonal shifts, especially in progesterone and estrogen, which are linked to increased frequency and severity of gingival inflammation. This inflammation typically peaks during the second or third month of pregnancy and interestingly resolves postpartum without lasting impacts on periodontal attachment levels (95, 104–106). The clinical inflammation observed during pregnancy is primarily driven by hormonal changes that modulate the immune response, increasing the production of pro-inflammatory cytokines (e.g., IL-1β, IL-6, IL-8, and TNF-α) and shifting the Th1/Th2 cytokine balance toward a Th1 bias in gingival tissues (106–108). Elevated progesterone also boosts prostaglandin E2 synthesis, which enhances vascular permeability and intensifies gingival inflammation (109). Moreover, it also exacerbates gingival inflammation by disturbing the balance between MMP-8 and MMP-9 and their tissue inhibitor TIMP-1, leading to increased MMP-8/TIMP-1 and MMP-9/TIMP-1 ratios (110). Additionally, these hormonal changes significantly transform the oral microbiome, increasing the prevalence of key periodontopathogens such as *Porphyromonas gingivalis* (*P. gingivalis*), *P. intermedia*, *Tannerella denticola*, *Aggregatibacter actinomycetemcomitans*, *Parvimonas micra*, *Neisseria*, *Treponema*, and *Fusobacterium*, which heightens the susceptibility to periodontal inflammation during pregnancy (107, 111–115).

Menopause is characterized by the cessation of ovarian activity and has profound systemic health implications, including periodontal health. Postmenopausal women, particularly those experiencing early menopause, often exhibit increased serum high-sensitivity C-reactive protein levels, greater clinical attachment loss, and increased percentage of bleeding on probing (BOP) compared to premenopausal counterparts (116). These women face a higher risk of developing moderate to severe periodontitis and experience a higher incidence of tooth loss (117, 118). Although hormone replacement therapy has been shown to modestly reduce the incidence of periodontitis, its effectiveness is diminished when adjusted for confounding factors such as age, body mass index, and smoking status (119).

### Gender-affirming hormone replacement therapy, immune response, and periodontal health in transgender populations

#### The role of GAHT in immunity

GAHT is a pivotal component in the transition process for transgender and non-binary individuals, aiming to align their physical appearance with their gender identity. This treatment strategy involves the careful selection and administration of hormones to either feminize or masculinize the body. Transgender women often receive a combination of estrogens, such as 17-β estradiol, and antiandrogens to markedly decrease testosterone levels (13). Conversely, transgender men and some non-binary individuals seeking masculinization receive testosterone therapy (13), which notably increases testosterone levels up to 20-fold, aligning them with the typical range seen in cisgender men (120). Not just a temporary intervention, GAHT frequently becomes a lifelong healthcare commitment, continuously supporting the individual’s gender identity and overall well-being.

The transformative role of GAHT extends beyond physical changes, significantly impacting the lives of transgender and non-binary individuals. However, the effects of GAHT on the immune system represent a less explored frontier. Emerging research begins to shed light on how hormonal changes induced by GAHT affect immunological functions. A pioneering study by Sellau et al. (121) specifically examined the impact of testosterone on cytokine expression in transgender men, revealing a significant increase in pro-inflammatory cytokines and chemokines such as TNF-α, IL-1*α*, CXCL1, and CCL2 over 200 days of testosterone therapy. These changes were directly linked to the rising plasma levels of testosterone and its metabolite, DHT, underscoring the significant role of testosterone in influencing immune responses (121). To contextualize these findings, it is important to compare them with observations from *in vitro* and *in vivo* studies, as well as clinical data on cis men under androgenic anabolic steroid (AAS) abuse. High doses of AAS in murine and *in vitro* studies have been shown to increase the production of pro-inflammatory cytokines such as IL-1 and TNF-α, while decreasing IL-4, IL-5, and IFNs (122, 123). Additionally, clinical data reveal that young male AAS users exhibit higher concentrations of IL-1 and IL-6 compared to non-users, although no significant differences in TNF-α levels were observed among groups (124). Interestingly, despite the increased pro-inflammatory cytokines, IL-10 levels were also higher in AAS users, suggesting a potential balancing effect on the pro/anti-inflammatory profile (124). These comparative insights highlight the complexity of testosterone's impact on the immune system. While transgender men on GAHT show increased pro-inflammatory markers similar to AAS users, the specific cytokine profile variations and the potential modulatory role of anti-inflammatory cytokines like IL-10 warrant further investigation.

Expanding on these findings, recent research has delved deeper into the immunological implications of GAHT, focusing particularly on the regulation of B cell-activating factor (BAFF) and TNF-α in a diverse cohort comprised of cisgender men and women, as well as transgender men and women who had undergone hormone therapy for a minimum of three years (125). The study unveiled a significant interaction between BAFF serum levels and sex hormones, revealing that both cisgender and transgender women have significantly higher BAFF levels compared to male participants, irrespective of their transgender or cisgender status (125). Additionally, this study shed light on the differential response of peripheral blood cells to LPS, revealing that LPS-induced TNF-α production was markedly increased in both cisgender and transgender men, indicating a gender-related difference in the immune system’s reaction to inflammatory stimuli (125). Building on these findings, a subsequent *in vivo* study assessed the impact of testosterone GAHT on immune cell dynamics in female mice (126). Testosterone administration to female C57BL/6J mice over eight weeks resulted in physiological changes consistent with clinical observations in humans undergoing GAHT (i.e. increased lean body mass and reduction in perigonadal fat mass) (126). Notably, the testosterone treatment led to a significant increase in peripheral Th17 cells (126).

The exploration into the effects of GAHT on the immune system underscores the complex modulation of immune responses by sex hormones. While research into the impacts of estrogen-based GAHT for transgender women and testosterone-based GAHT for transgender men has advanced, significant gaps remain. These gaps hinder our comprehensive understanding of GAHT's implications for periodontal health. We can speculate that the increases in TNF-α, IL-1α, CXCL1, CCL2, and Th17 responses following testosterone GAHT therapy (121–126) could increase inflammation in periodontal tissues, potentially elevating periodontitis risk (121–126). Conversely, the elevated serum levels of B cell activating factor (BAFF) observed in transgender women on GAHT may indicate a hormone-related modulation of B cell function, which plays a critical role in inflammatory responses and alveolar bone loss (125). Pre-clinical studies further illuminate this dynamic. For example, testosterone supplementation in female rats did not markedly alter ligature-induced bone loss, yet it reduced inflammatory mediators such as MIP-1α, IL-1α, IL-1β, IL-10, and RANTES (85). These changes, however, could be an indirect role of testosterone, potentially through its conversion to estradiol. Supporting this, a subsequent study that combined testosterone supplementation with an aromatase inhibitor to prevent estrogen signaling in female rats demonstrated increased osteoclast activity and ligature-induced bone loss, accompanied by elevated PGE2 and reduced IL-2 levels in periodontal tissues (84). These findings indicate that the effects of supplemental testosterone on periodontal health may be partially mediated through its estrogenic metabolites. Additionally, although direct data on estrogen supplementation and ligature-induced bone loss in male rats are lacking, related research on the influence of estrogen on bone modeling provides relevant insights. A study evaluating the effects of estrogen on the mandibular condylar bone in orchiectomized male mice showed that estradiol supplementation after orchiectomy mitigated the increase in TRAP-positive cells and reversed the loss of trabecular bone volume in the condylar head (127), suggesting a protective role of estrogen in bone preservation, which might extend implications for periodontal health in GAHT contexts.

The limited number of studies investigating the effects of GAHT on immune responses in humans is evident. Table 1 summarizes the findings from the only two studies conducted on this topic, highlighting the need for more research in this area.

**Table 1:** Key findings related to GAHT and immune responses in transgender humans.

Study	Population	Hormonal treatment	Key findings
Sellau et al. (2020) (121)	• Transgender men	Testosterone	Significant increase in pro-inflammatory cytokines (TNF-α, IL-1α, CXCL1, CCL2) after 200 days of therapy. Direct correlation with plasma testosterone and 5α-dihydrotestosterone levels.
Tsatsanis et al. (2023) (125)	• Transgender men • Transgender women • Cisgender men, • Cisgender women	Transgender men: testosterone Transgender women: estrogen and antiandrogen	Transgender women and cisgender women had higher BAFF levels irrespective of their sex chromosome. LPS-induced TNF-α production was higher in cisgender men and transgender men compared to cisgender women and transgender women.

### Summary and future perspectives

**Figure 1:**
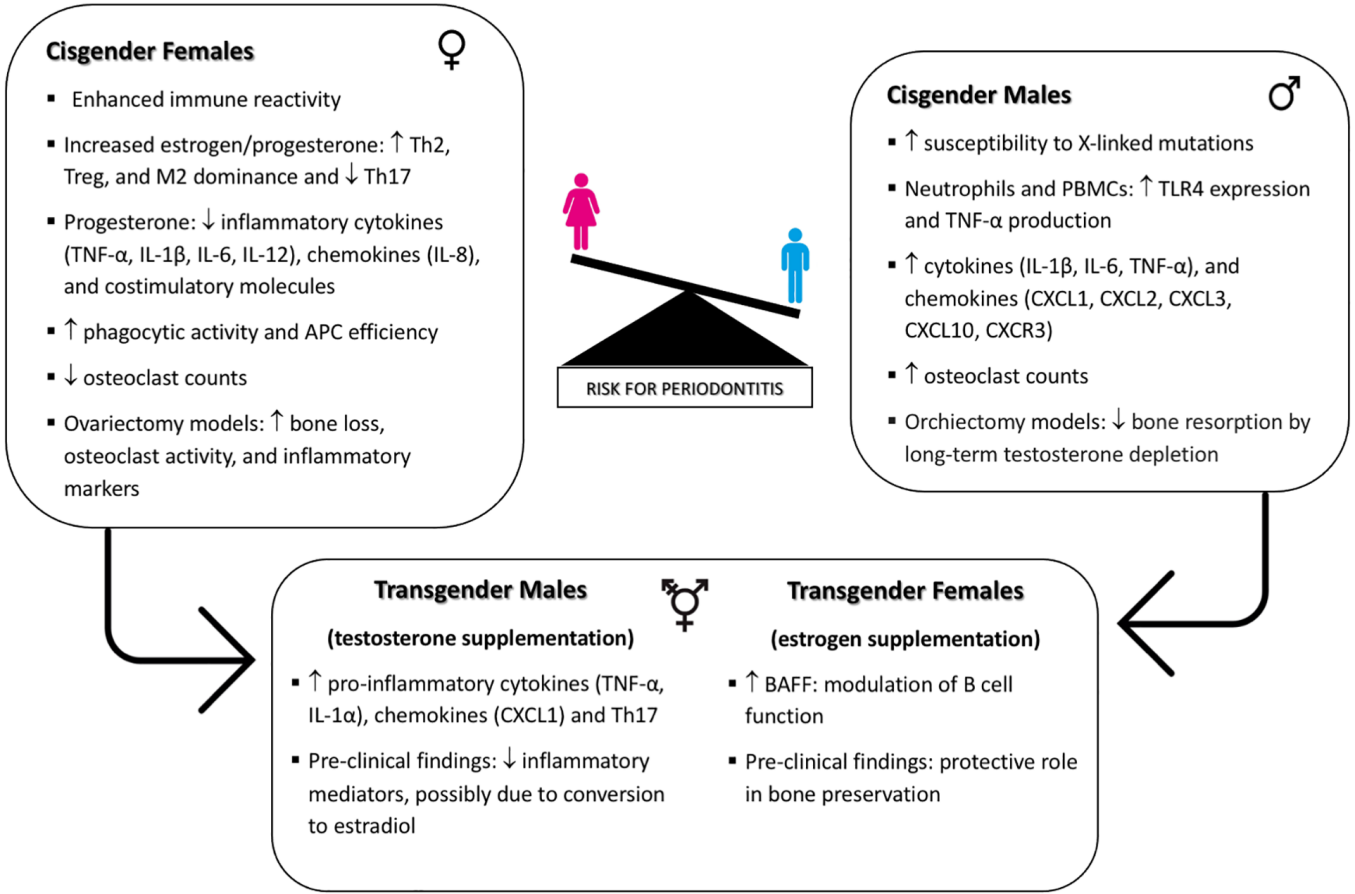
Interplay of biological sex, GAHT, and immune regulation in periodontal disease susceptibility.

In this review, we explore the intricate relationship between biological sex, GAHT, and periodontal health, elucidating the underlying mechanisms that might influence periodontal disease susceptibility and progression. A model is illustrated in the accompanying Figure 1. Sexual dimorphism significantly impacts immune function, as evidenced by the differential expression of immune-related genes on the X and Y chromosomes and the modulation of immune responses by sex hormones. Cisgender females exhibit enhanced immune reactivity due to the presence of two X chromosomes, which contribute to a more robust immune response. Elevated levels of estrogen and progesterone in cisgender females influence immune regulation by shifting responses towards Th2, Treg, and M2 dominance while suppressing Th1, Th17, and M1 cell activities. Progesterone also inhibits the production of inflammatory cytokines such as TNF-α, IL-1β, IL-6, and IL-12, as well as chemokines like IL-8, and costimulatory molecules including CD40, CD80, and CD86. This hormonal environment enhances phagocytic activity and antigen presentation efficiency by antigen-presenting cells and lowers osteoclast counts, underscoring the protective role of physiological levels of estrogen and progesterone in periodontal health. In contrast, cisgender males, possessing a single X chromosome, are more susceptible to X-linked gene mutations, which can influence immune function. Cisgender males exhibit enhanced TLR4 expression in neutrophils and peripheral blood mononuclear cells (PBMCs), leading to higher TNF-α production while inhibiting the microbicidal activities of innate immune cells. Elevated levels of chemokines (CXCL1, CXCL2, CXCL3) and cytokines (IL-1β, IL-6, TNF-α) are observed, along with higher expression of CXCR3 and CXCL10, which increase IFN-γ production and macrophage activation. These findings, associated with higher osteoclast counts in cisgender males, are likely to increase their susceptibility to periodontal diseases.

GAHT introduces additional complexity in immune modulation for transgender individuals. In transgender females, elevated serum levels of B cell activating factor (BAFF) suggest a hormone-related modulation of B cell function, influencing inflammatory responses and alveolar bone loss. Studies on estrogen supplementation in male mice indicate a protective role in bone preservation, which may extend to periodontal health in transgender women. Conversely, testosterone therapy in transgender males increases pro-inflammatory cytokines (e.g., TNF-α, IL-1α, CXCL1) and Th17 cells, potentially heightening inflammation in periodontal tissues and elevating periodontitis risk. However, pre-clinical findings indicate that while testosterone supplementation in female rats reduces inflammatory mediators, this effect may be due to the conversion of testosterone to estradiol. This emphasizes the nuanced impact of GAHT on periodontal health, highlighting the complexity where exogenous hormones may be metabolized differently across sexes and/or have varied impacts due to differences in receptor expression.

The exploration of the immunological impacts of GAHT presents a promising yet largely untapped frontier in personalized medicine. Future research should prioritize comprehensive, long-term studies to elucidate the intricate mechanisms by which GAHT influences immune responses and periodontal health in transgender individuals. Given the growing population seeking gender-affirming care, it is crucial to investigate how hormonal variations affect susceptibility to autoimmune and inflammatory conditions, including periodontitis. Expanding research to include diverse cohorts, particularly those that have been historically underrepresented, will ensure that findings are representative and applicable to all individuals undergoing GAHT. Furthermore, addressing barriers to healthcare access for transgender individuals, such as discrimination, lack of provider knowledge, and socioeconomic disparities, is essential for facilitating participation in research and improving health outcomes. By addressing these gaps, we can pave the way for more effective, personalized healthcare strategies that account for the unique immunological profiles of transgender patients, ultimately improving their overall health outcomes and quality of life.

## References

[1] Janakiram C Dye BA. A public health approach for prevention of periodontal disease. Periodontol 2000. 2020;8:202–14. 10.1111/prd.12337 PMC7457923 32844412

[2] Hajishengallis G. Immunomicrobial pathogenesis of periodontitis: keystones, pathobionts, and host response. Trends Immunol. 2014a;35:3–11. 10.1016/j.it.2013.09.001 24269668 PMC3947349

[3] Bartold PM Van Dyke TE. Host modulation: controlling the inflammation to control the infection. Periodontol 2000. 2017;75:317–29. 10.1111/prd.12169 28758299

[4] Loos BG Van Dyke TE. The role of inflammation and genetics in periodontal disease. Periodontol 2000. 2020;83:26–39. 10.1111/prd.12297 32385877 PMC7319430

[5] Liu Y Yu Y Nickel JC Iwasaki LR Duan P Simmer-Beck M Brown L. Gender differences in the association of periodontitis and type 2 diabetes. Int Dent J. 2018; 68:433–40. 10.1111/idj.12399 29786140 PMC9379021

[6] Klein SL Flanagan KL. Sex differences in immune responses. Nat Rev Immunol. 2016;16:626–38. 10.1038/nri.2016.90 27546235

[7] Berletch JB Yang F Xu J Carrel L Disteche CM. Genes that escape from X inactivation. Hum Genet. 2011;130:237–45. 10.1007/s00439-011-1011-z 21614513 PMC3136209

[8] Laffont S Rouquié N Azar P Seillet C Plumas J Aspord C X-chromosome complement and estrogen receptor signaling independently contribute to the enhanced TLR7-mediated IFN-α production of plasmacytoid dendritic cells from women. J Immunol. 2014;193:5444–52. 10.4049/jimmunol.1303400 25339659

[9] Walecki M Eisel F Klug J Baal N Paradowska-Dogan A Wahle E Androgen receptor modulates Foxp3 expression in CD4 + CD25 + Foxp3 + regulatory T-cells. Mol Biol Cell. 2015;26:2845–57. 10.1091/mbc.E14-08-1323 26063731 PMC4571343

[10] Markle JG Fish EN. SeXX matters in immunity. Trends Immunol. 2014;35:97–104. 10.1016/j.it.2013.10.006 24239225

[11] Wilkinson NM Chen HC Lechner MG Su MA. Sex differences in immunity. Annu Rev Immunol. 2022;40:75–94. 10.1146/annurev-immunol-101320-125133 34985929 PMC9805670

[12] Moulton VR. Sex hormones in acquired immunity and autoimmune disease. Front Immunol. 2018;9:2279. 10.3389/fimmu.2018.02279 30337927 PMC6180207

[13] Hembree WC Cohen-Kettenis PT Gooren L Hannema SE Meyer WJ Murad MH Endocrine treatment of gender-dysphoric/gender-incongruent persons: an endocrine society clinical practice guideline. J Clin Endocrinol Metab. 2017;102:3869–903. 10.1210/jc.2017-01658 28945902

[14] Schulz SL. The informed consent model of transgender care: an alternative to the diagnosis of gender dysphoria. J Humanist Psychol. 2018;58:72–92. 10.1177/002216781774

[15] White AA Lin A Bickendorf X Cavve BS Moore JK Siafarikas A Strickland DH Leffler J. Potential immunological effects of gender-affirming hormone therapy in transgender people—an unexplored area of research. Ther Adv Endocrinol Metab. 2022;13:20420188221139612. 10.1177/20420188221139612 36533187 PMC9747891

[16] Hajishengallis G. The inflammophilic character of the periodontitis-associated microbiota. Mol Oral Microbiol. 2014b;29:248–57. 10.1111/omi.12065 24976068 PMC4232466

[17] Kantarci A Oyaizu K Van Dyke TE. Neutrophil-mediated tissue injury in periodontal disease pathogenesis: findings from localized aggressive periodontitis. J Periodontol. 2003;74:66–75. 10.1902/jop.2003.74.1.66 12593599

[18] Dutzan N Konkel JE Greenwell-Wild T Moutsopoulos NM. Characterization of the human immune cell network at the gingival barrier. Mucosal Immunol. 2016;9:1163–72. 10.1038/mi.2015.136 26732676 PMC4820049

[19] Sloniak MC Lepique AP Nakao LYS Villar CC. Alterations in macrophage polarization play a key role in control and development of periodontal diseases. J Indian Soc Periodontol. 2023;27:578–82. 10.4103/jisp.jisp_75_23 38434507 PMC10906788

[20] Campbell L Millhouse E Malcolm J Culshaw S. T cells, teeth and tissue destruction—what do T cells do in periodontal disease? Mol Oral Microbiol. 2016;31:445–56. 10.1111/omi.12144 26505640

[21] Feng Y Chen Z Tu SQ Wei JM Hou YL Kuang ZL Role of interleukin-17A in the pathomechanisms of periodontitis and related systemic chronic inflammatory diseases. Front Immunol. 2022;13:862415. 10.3389/fimmu.2022.862415 35371044 PMC8968732

[22] Monasterio G Castillo F Ibarra JP Guevara J Rojas L Alvarez C Alveolar bone resorption and Th1/Th17-associated immune response triggered during Aggregatibacter actinomycetemcomitans-induced experimental periodontitis are serotype-dependent. J Periodontol. 2018;89:1249–61. 10.1002/JPER.17-0563 30030845

[23] Cafferata EA Castro-Saavedra S Fuentes-Barros G Melgar-Rodríguez S Rivera F Carvajal P Boldine inhibits the alveolar bone resorption during ligature-induced periodontitis by modulating the Th17/Treg imbalance. J Periodontol. 2021;92:123–36. 10.1002/JPER.20-0055 32490537

[24] Gaffen SL Hajishengallis G. A new inflammatory cytokine on the block: re-thinking periodontal disease and the Th1/Th2 paradigm in the context of Th17 cells and IL-17. J Dent Res. 2008;87:817–28. 10.1177/154405910808700908 18719207 PMC2692983

[25] Jagannathan M Hasturk H Liang Y Shin H Hetzel JT Kantarci A TLR cross-talk specifically regulates cytokine production by B cells from chronic inflammatory disease patients. J. Immunol. 2009;183:7461–70. 10.4049/jimmunol.0901517 19917698 PMC2851147

[26] Artese L Simon MJ Piattelli A Ferrari DS Cardoso LA Faveri M Immunohistochemical analysis of inflammatory infiltrate in aggressive and chronic periodontitis: a comparative study. Clin Oral Investig. 2011;15:233–40. 10.1007/s00784-009-0374-1 20058159

[27] Seymour GJ Powell RN Davies WI. The immunopathogenesis of progressive chronic inflammatory periodontal disease. J Oral Pathol. 1979;8:249–65. 10.1111/j.1600-0714.1979.tb01826.x 119837

[28] Mahanonda R Champaiboon C Subbalekha K Sa-Ard-Iam N Rattanathammatada W Thawanaphong S Human memory B cells in healthy gingiva, gingivitis, and periodontitis. J Immunol. 2016;197:715–25. 10.4049/jimmunol.1600540 27335500

[29] Kawai T Matsuyama T Hosokawa Y Makihira S Seki M Karimbux NY B and T lymphocytes are the primary sources of RANKL in the bone resorptive lesion of periodontal disease. Am J Pathol. 2006;169:987–98. 10.2353/ajpath.2006.060180 16936272 PMC1698808

[30] Kanzaki H Makihira S Suzuki M Ishii T Movila A Hirschfeld J Soluble RANKL cleaved from activated lymphocytes by TNF-alpha-converting enzyme contributes to osteoclastogenesis in periodontitis. J Immunol. 2016;197:3871–83. 10.4049/jimmunol.1601114 27815441 PMC5120874

[31] Zouali M. The emerging roles of B cells as partners and targets in periodontitis. Autoimmunity. 2017;50:61–70. 10.1080/08916934.2016.1261841 28013554

[32] Hascoët E Blanchard F Blin-Wakkach C Guicheux J Lesclous P Cloitre A. New insights into inflammatory osteoclast precursors as therapeutic targets for rheumatoid arthritis and periodontitis. Bone Res. 2023;11:26. 10.1038/s41413-023-00257-w 37217496 PMC10203317

[33] Libert C Dejager L Pinheiro I. The X chromosome in immune functions: when a chromosome makes the difference. Nat Rev Immunol. 2010;10:594–604. 10.1038/nri2815 20651746

[34] Migeon BR. X-linked diseases: susceptible females. Genet Med. 2020;22:1156–74. 10.1038/s41436-020-0779-4 32284538 PMC7332419

[35] Fish E. The X-files in immunity: sex-based differences predispose immune responses. Nat Rev Immunol. 2008;8:737–44. 10.1038/nri2394 18728636 PMC7097214

[36] Schurz H Salie M Tromp G Hoal EG Kinnear CJ Möller M. The X chromosome and sex-specific effects in infectious disease susceptibility. Hum Genomics. 2019;13:2. 10.1186/s40246-018-0185-z 30621780 PMC6325731

[37] Di Palo A Siniscalchi C Salerno M Russo A Gravholt CH Potenza N. What microRNAs could tell us about the human X chromosome. Cell Mol Life Sci. 2020;77:4069–80. 10.1007/s00018-020-03526-7 32356180 PMC7854456

[38] Teuscher C Noubade R Spach K McElvany B Bunn JY Fillmore PD Evidence that the Y chromosome influences autoimmune disease in male and female mice. Proc Natl Acad Sci U S A. 2006;103:8024–9. 10.1073/pnas.0600536103 16702550 PMC1472423

[39] Spach KM Blake M Bunn JY McElvany B Noubade R Blankenhorn EP Cutting edge: the Y chromosome controls the age-dependent experimental allergic encephalomyelitis sexual dimorphism in SJL/J mice. J Immunol. 2009;182:1789–93. 10.4049/jimmunol.0803200 19201829 PMC2658649

[40] Krementsov DN Case LK Dienz O. Genetic variation in chromosome Y regulates susceptibility to influenza A virus infection. Proc Natl Acad Sci U S A. 2017;114:3491–6. 10.1073/pnas.1620889114 28242695 PMC5380050

[41] Case LK Toussaint L Moussawi M Roberts B Saligrama N Brossay L Chromosome Y regulates survival following murine Coxsackievirus B3 infection. G3. 2012;2:115–21. 10.1534/g3.111.001610 22384388 PMC3276194

[42] Schiffer L Barnard L Baranowski ES Gilligan LC Taylor AE Arlt W Human steroid biosynthesis, metabolism and excretion are differentially reflected by serum and urine steroid metabolomes: a comprehensive review. J Steroid Biochem Mol Biol. 2019;194:105439. 10.1016/j.jsbmb.2019.105439 31362062 PMC6857441

[43] Królik M Milnerowicz H. The effect of using estrogens in the light of scientific research. Adv Clin Exp Med. 2012;21:535–43. 23240460

[44] Kovats S. Estrogen receptors regulate innate immune cells and signaling pathways. Cell Immunol. 2015;294:63–9. 10.1016/j.cellimm.2015.01.018 25682174 PMC4380804

[45] Keselman A Fang X White PB Heller NM. Estrogen signaling contributes to sex differences in macrophage polarization during asthma. J Immunol. 2017;199:1573–83. 10.4049/jimmunol.1601975 28760880 PMC5576568

[46] Wu WF Tan XJ Dai YB Krishnan V Warner M Gustafsson JA. Targeting estrogen receptor *β* in microglia and T cells to treat experimental autoimmune encephalomyelitis. Proc Natl Acad Sci U S A. 2013;110:3543–8. 10.1073/pnas.1300313110 23401502 PMC3587193

[47] Molloy EJ O'Neill AJ Grantham JJ Sheridan-Pereira M Fitzpatrick JM Webb DW Sex-specific alterations in neutrophil apoptosis: the role of estradiol and progesterone. Blood. 2003;102:2653–9. 10.1182/blood-2003-02-0649 12791649

[48] Goodman WA Bedoyan SM Havran HL Richardson B Cameron MJ Pizarro TT. Impaired estrogen signaling underlies regulatory T cell loss-of-function in the chronically inflamed intestine. Proc Natl Acad Sci U S A. 2020;117:17166–76. 10.1073/pnas.2002266117 32632016 PMC7382259

[49] Kanda N Tamaki K. Estrogen enhances immunoglobulin production by human PBMCs. J Allergy Clin Immunol. 1999;103:282–8. 10.1016/s0091-6749(99)70503-8 9949320

[50] Lü FX Abel K Ma Z Rourke T Lu D Torten J The strength of B cell immunity in female rhesus macaques is controlled by CD8+ T cells under the influence of ovarian steroid hormones. Clin Exp Immunol. 2002;128:10–20. 10.1046/j.1365-2249.2002.01780.x 11982585 PMC1906365

[51] Butts CL Shukair SA Duncan KM Bowers E Horn C Belyavskaya E Progesterone inhibits mature rat dendritic cells in a receptor-mediated fashion. Int Immunol. 2007;3:287–96. 10.1093/intimm/dxl145 17289656

[52] Jones LA Kreem S Shweash M Paul A Alexander J Roberts CW. Differential modulation of TLR3- and TLR4-mediated dendritic cell maturation and function by progesterone. J Immunol. 2010;185:4525–34. 10.4049/jimmunol.0901155 20844199

[53] Grandi G Mueller M Bersinger N Papadia A Nirgianakis K Cagnacci A Progestin suppressed inflammation and cell viability of tumor necrosis factor-α-stimulated endometriotic stromal cells. Am J Reprod Immunol. 2016;76:292–8. 10.1111/aji.12552 27515307

[54] Hierweger AM Engler JB Friese MA Reichardt HM Lydon J DeMayo F Progesterone modulates the T-cell response via glucocorticoid receptor-dependent pathways. Am J Reprod Immunol. 2019;81:e13084. 10.1111/aji.13084 30604567 PMC7457140

[55] Lee JH Ulrich B Cho J Park J Kim CH. Progesterone promotes differentiation of human cord blood fetal T cells into T regulatory cells but suppresses their differentiation into Th17 cells. J Immunol. 2011;187:1778–87. 10.4049/jimmunol.1003919 21768398 PMC3155957

[56] Miyaura H Iwata M. Direct and indirect inhibition of Th1 development by progesterone and glucocorticoids. J Immunol. 2002;168:1087–94. 10.4049/jimmunol.168.3.1087 11801642

[57] Chuang KH Altuwaijri S Li G Lai JJ Chu CY Lai KP Neutropenia with impaired host defense against microbial infection in mice lacking androgen receptor. J Exp Med. 2009;206:1181–99. 10.1084/jem.20082521 19414555 PMC2715023

[58] Marin DP Bolin AP dos Santos RCM Curi R Otton R. Testosterone suppresses oxidative stress in human neutrophils. Cell Biochem Funct. 2010;28:394–402. 10.1002/cbf.1669 20589735

[59] Lotter H Helk E Bernin H Jacobs T Prehn C Adamski J Testosterone increases susceptibility to amebic liver abscess in mice and mediates inhibition of IFN*γ* secretion in natural killer T cells. PLoS One. 2013;8:e55694. 10.1371/journal.pone.0055694 23424637 PMC3570563

[60] Er-Lukowiak M Hänzelmann S Rothe M Moamenpour DT Hausmann F Khatri R Testosterone affects type I/type II interferon response of neutrophils during hepatic amebiasis. Front Immunol. 2023;14:1279245. 10.3389/fimmu.2023.1279245 38179044 PMC10764495

[61] Altuwaijri S Chuang KH Lai KP Lai JJ Lin HY Young FM Susceptibility to autoimmunity and B cell resistance to apoptosis in mice lacking androgen receptor in B cells. Mol Endocrinol. 2009;23:444–53. 10.1210/me.2008-0106 19164450 PMC2667704

[62] Zhu ML Bakhru P Conley B Nelson JS Free M Martin A Sex bias in CNS autoimmune disease mediated by androgen control of autoimmune regulator. Nat Commun. 2016;7:11350. 10.1038/ncomms11350 27072778 PMC5512610

[63] Wilson JD. The role of 5alpha-reduction in steroid hormone physiology. Reprod Fertil Dev. 2001;13:673–8. 10.1071/rd01074 11999320

[64] Fouad Mansour M Pelletier M Boulet MM Mayrand D Brochu G Lebel S Oxidative activity of 17β-hydroxysteroid dehydrogenase on testosterone in male abdominal adipose tissues and cellular localization of 17β-HSD type 2. Mol Cell Endocrinol. 2015;;414:168–76. 10.1016/j.mce.2015.06.016 26123590

[65] Aomatsu M Kato T Kasahara E Kitagawa S. Gender difference in tumor necrosis factor-α production in human neutrophils stimulated by lipopolysaccharide and interferon-γ. Biochem Biophys Res Commun. 2013;441:220–5. 10.1016/j.bbrc.2013.10.042 24140406

[66] Asai K Hiki N Mimura Y Ogawa T Unou K Kaminishi M. Gender differences in cytokine secretion by human peripheral blood mononuclear cells: role of estrogen in modulating LPS-induced cytokine secretion in an ex vivo septic model. Shock. 2001;16:340–3. 10.1097/00024382-200116050-00003 11699070

[67] D'Agostino P Milano S Barbera C Di Bella G La Rosa M Ferlazzo V Sex hormones modulate inflammatory mediators produced by macrophages. Ann N Y Acad Sci. 1999;876:426–9. 10.1111/j.1749-6632.1999.tb07667.x 10415638

[68] Rettew JA Huet-Hudson YM Marriott I. Testosterone reduces macrophage expression in the mouse of toll-like receptor 4, a trigger for inflammation and innate immunity. Biol Reprod. 2008;78:432–7. 10.1095/biolreprod.107.063545 18003947

[69] Sciarra F Campolo F Franceschini E Carlomagno F Venneri MA. Gender-specific impact of sex hormones on the immune system. Int J Mol Sci. 2023;24:6302. 10.3390/ijms24076302 37047274 PMC10094624

[70] Spitzer JA. Gender differences in some host defense mechanisms. Lupus. 1999;8:380–3. 10.1177/096120339900800510 10455517

[71] Weinstein Y Ran S Segal S. Sex-associated differences in the regulation of immune responses controlled by the MHC of the mouse. J Immunol. 1984;132:656–61. 10.4049/jimmunol.132.2.656 6228595

[72] Wang C Dehghani B Li Y Kaler LJ Vandenbark AA Offner H. Oestrogen modulates experimental autoimmune encephalomyelitis and interleukin-17 production via programmed death 1. Immunology. 2009;126:329–35. 10.1111/j.1365-2567.2008.03051.x 19302141 PMC2669813

[73] Anbinder AL Moraes RM Lima GMG Oliveira FE Campos DRC Rossoni RD Periodontal disease exacerbates systemic ovariectomy-induced bone loss in mice. Bone. 2016;83:241–7. 10.1016/j.bone.2015.11.014 26620086

[74] Dai J Ma Y Shi M Cao Z Zhang Y Miron RJ. Initial changes in alveolar bone volume for sham-operated and ovariectomized rats in ligature-induced experimental periodontitis. Clin Oral Investig. 2016;20:581–8. 10.1007/s00784-015-1531-3 26179986

[75] Johnson RB Gilbert JA Cooper RC Dai X Newton BI Tracy RR Alveolar bone loss one year following ovariectomy in sheep. J Periodontol. 1997;68:864–71. 10.1902/jop.1997.68.9.864 9379331

[76] Fujita S Kikuchi T Sobue T Suzuki M Koide M Noguchi T. Lipopolysaccharide-mediated enhancement of bone metabolism in estrogen-deficient mice. J Periodontol. 2008;79:2173–81. 10.1902/jop.2008.070127 18980527

[77] Takayanagi H Ogasawara K Hida S Chiba T Murata S Sato K T-cell-mediated regulation of osteoclastogenesis by signaling cross-talk between RANKL and IFN-gamma. Nature. 2000;30;408:600–5. 10.1038/35046102 11117749

[78] Tanaka M Ejiri S Toyooka E Kohno S Ozawa H. Effects of ovariectomy on trabecular structures of rat alveolar bone. J Periodontal Res. 2002;37:161–5. 10.1034/j.1600-0765.2002.01601.x 12009186

[79] Duarte PM Gonçalves PF Sallum AW Sallum EA Casati MZ, Humberto Nociti F Jr. Effect of an estrogen-deficient state and its therapy on bone loss resulting from an experimental periodontitis in rats. J Periodontal Res. 2004;39:107–10. 10.1111/j.1600-0765.2004.00714.x 15009518

[80] Nebel D Bratthall G Warfvinge G Nilsson BO. Effects of ovariectomy and aging on tooth attachment in female mice assessed by morphometric analysis. Acta Odontol Scand. 2009;67:8–12. 10.1080/00016350802443474 18923970

[81] Lee DJ Wu L Shimono M Piao Z Green DW Lee JM Differential mechanism of periodontitis progression in postmenopause. Front Physiol. 2018;14;9:1098. 10.3389/fphys.2018.01098 PMC6113945 30246792

[82] Steffens JP Coimbra LS Rossa C Jr Kantarci A Van Dyke TE Spolidorio LC. Androgen receptors and experimental bone loss—an *in vivo* and *in vitro* study. Bone. 2015;81:683–90. 10.1016/j.bone.2015.10.001 26450018 PMC4641040

[83] Pelegrin ÁF de Paiva Gonçalves V Carvalho JS Spolidorio DMP Spolidorio LC. Testosterone replacement relieves ligature-induced periodontitis by mitigating inflammation, increasing pro-resolving markers and promoting angiogenesis in rats: a preclinical study. Arch Oral Biol. 2023;146:105605. 10.1016/j.archoralbio.2022.105605 36521281

[84] Steffens JP Valenga HM Santana LCL Albaricci MCDC Kantarci A Spolidorio LC. Role of testosterone and androgen receptor in periodontal disease progression in female rats. J Periodontol. 2020;91:545–53. 10.1002/JPER.19-0099 31389012

[85] Steffens JP Santana LCL Pitombo JCP Ribeiro DO Albaricci MCC Warnavin SVSC The role of androgens on periodontal repair in female rats. J Periodontol. 2018;89:486–95. 10.1002/JPER.17-0435 29683499

[86] de Paiva Gonçalves V Ortega AAC Steffens JP Spolidorio DMP Rossa C Spolidorio LC. Long-term testosterone depletion attenuates inflammatory bone resorption in the ligature-induced periodontal disease model. J Periodontol. 2018;89:466–75. 10.1002/JPER.17-0457 29683497

[87] Valerio MS Kirkwood KL. Sexual dimorphism in immunity to oral bacterial diseases: intersection of neutrophil and osteoclast pathobiology. J Dent Res. 2018;97:1416–23. 10.1177/0022034518798825 30205018 PMC6262266

[88] Valerio MS Basilakos DS Kirkpatrick JE Chavez M Hathaway-Schrader J Herbert BA Sex-based differential regulation of bacterial-induced bone resorption. J Periodontal Res. 2017;52:377–87. 10.1111/jre.12401 27509894 PMC5303566

[89] Mantovani A Cassatella MA Costantini C Jaillon S. Neutrophils in the activation and regulation of innate and adaptive immunity. Nat Rev Immunol. 2011;11:519–31. 10.1038/nri3024 21785456

[90] Sadik CD Kim ND Luster AD. Neutrophils cascading their way to inflammation. Trends Immunol. 2011;32:452–60. 10.1016/j.it.2011.06.008 21839682 PMC3470857

[91] Havens AM Chiu E Taba M Wang J Shiozawa Y Jung Y Stromal-derived factor-1alpha (CXCL12) levels increase in periodontal disease. J Periodontol. 2008;79:845–53. 10.1902/jop.2008.070514 18454663 PMC2582372

[92] Scotland RS Stables MJ Madalli S Watson P Gilroy DW. Sex differences in resident immune cell phenotype underlie more efficient acute inflammatory responses in female mice. Blood. 2011;118:5918–27. 10.1182/blood-2011-03-340281 21911834 PMC5363818

[93] Garlet GP Martins W Jr Ferreira BR Milanezi CM Silva JS. Patterns of chemokines and chemokine receptors expression in different forms of human periodontal disease. J Periodontal Res. 2003;38:210–7. 10.1034/j.1600-0765.2003.02012.x 12608917

[94] Machtei EE Mahler D Sanduri H Peled M. The effect of menstrual cycle on periodontal health. J Periodontol. 2004;75:408–12. 10.1902/jop.2004.75.3.408 15088879

[95] Romandini M Shin HS Romandini P Laforí A Cordaro M. Hormone-related events and periodontitis in women. J Clin Periodontol. 2020;47:429–41. 10.1111/jcpe.13248 31912529

[96] Aufdemorte TB Sheridan PJ. Nuclear uptake of sex steroids in gingiva of the baboon. J Periodontol. 1981;52:430–4. 10.1902/jop.1981.52.8.430 6943329

[97] Jönsson D Andersson G Ekblad E Liang M Bratthall G Nilsson BO. Immunocytochemical demonstration of estrogen receptor beta in human periodontal ligament cells. Arch Oral Biol. 2004;49:85–8. 10.1016/s0003-9969(03)00198-5 14693201

[98] Mombelli A Gusberti FA van Oosten MA Lang NP. Gingival health and gingivitis development during puberty. A 4-year longitudinal study. J Clin Periodontol. 1989;16:451–6. 10.1111/j.1600-051x.1989.tb01674.x 2768539

[99] Khosravisamani M Maliji G Seyfi S Azadmehr A Abd Nikfarjam B Madadi S Effect of the menstrual cycle on inflammatory cytokines in the periodontium. J Periodontal Res. 2014;49:770–6. 10.1111/jre.12161 24673464

[100] Gusberti FA Mombelli A Lang NP Minder CE. Changes in subgingival microbiota during puberty. A 4-year longitudinal study. J Clin Periodontol. 1990;17:685–92. 10.1111/j.1600-051x.1990.tb01054.x 2262580

[101] Steffens JP Herrera BS Coimbra LS Stephens DN Rossa C Jr Spolidorio LC Kantarci A Van Dyke TE. Testosterone regulates bone response to inflammation. Horm Metab Res. 2014;46:193–200. 10.1055/s-0034-1367031 24526374 PMC4522923

[102] Steffens JP Wang X Starr JR Spolidorio LC Van Dyke TE Kantarci A. Associations between sex hormone levels and periodontitis in men: results from NHANES III. J Periodontol. 2015;86:1116–25. 10.1902/jop.2015.140530 26062840

[103] von Stein Cubas Warnavin S Valenga HM Costa TBC Chaves JDP Spolidorio LC Spolidorio DMP Feres M Soares GMS Steffens JP. Periodontal clinical status, microbial profile, and expression of interleukin-1β in men under androgenic anabolic steroids abuse. Clin Oral Investig. 2021;25:3567–75. 10.1007/s00784-020-03679-6 33179177

[104] Gürsoy M Pajukanta R Sorsa T Könönen E. Clinical changes in periodontium during pregnancy and post-partum. J Clin Periodontol. 2008;35:576–383. 10.1111/j.1600-051X.2008.01236.x 18430046

[105] Figuero E Carrillo-de-Albornoz A Martín C Tobías A Herrera D. Effect of pregnancy on gingival inflammation in systemically healthy women: a systematic review. J Clin Periodontol. 2013;40:457–73. 10.1111/jcpe.12053 23557432

[106] Wen X Fu X Zhao C Yang L Huang R. The bidirectional relationship between periodontal disease and pregnancy via the interaction of oral microorganisms, hormone and immune response. Front Microbiol. 2023;14:1070917. 10.3389/fmicb.2023.1070917 36778874 PMC9908602

[107] Carrillo-de-Albornoz A Figuero E Herrera D Cuesta P Bascones-Martínez A. Gingival changes during pregnancy: III. Impact of clinical, microbiological, immunological and socio-demographic factors on gingival inflammation. J Clin Periodontol. 2012;39:272–83. 10.1111/j.1600-051X.2011.01800.x 22092526

[108] Meriç P Silbereisen A Emingil G Öztürk VÖ Bostanci N. Clinical, oral immunological and microbiological shifts during and after pregnancy. Clin Oral Investig. 2023;28:60. 10.1007/s00784-023-05408-1 PMC10756889 38157038

[109] Perunovic NDj Rakic MM Nikolic LI Jankovic SM Aleksic ZM Plecas DV The association between periodontal inflammation and labor triggers (elevated cytokine levels) in preterm birth: a cross-sectional study. J Periodontol. 2016;87:248–56. 10.1902/jop.2015.150364 26447753

[110] Özgen Öztürk V Meriç P Sorsa T Tervahartiala T Bostanci N Nwhator SO Regulation of matrix metalloproteinases-8, -9 and endogenous tissue inhibitor-1 in oral biofluids during pregnancy and postpartum. Arch Oral Biol. 2021;124:105065. 10.1016/j.archoralbio.2021.105065 33556788

[111] Carrillo-de-Albornoz A Figuero E Herrera D Bascones-Martinez A. Gingival changes during pregnancy II. Influence of hormonal variations on the subgingival biofilm. J Clin Periodontol. 2010;37:230–40. 10.1111/j.1600-051X.2009.01514.x 20088983

[112] Kumar PS. Sex and the subgingival microbiome: do female sex steroids affect periodontal bacteria? Periodontol 2000. 2013;61:103–24. 10.1111/j.1600-0757.2011.00398.x 23240946

[113] Fujiwara N Tsuruda K Iwamoto Y Kato F Odaki T Yamane N Significant increase of oral bacteria in the early pregnancy period in Japanese women. J Investig Clin Dent. (2017) 8(1):1–8. 10.1111/jicd.12189 26345599

[114] Lin W Jiang W Hu X Gao L Ai D Pan H Ecological shifts of supragingival microbiota in association with pregnancy. Front Cell Infect Microbiol. 2018;8:24. 10.3389/fcimb.2018.00024 29497601 PMC5819318

[115] Jang H Patoine A Wu TT Castillo DA Xiao J. Oral microflora and pregnancy: a systematic review and meta-analysis. Sci Rep. 2021;11:16870. 10.1038/s41598-021-96495-1 34413437 PMC8377136

[116] Sharma N Sharma RK Tewari S Chauhan M Bhatia A. Association of periodontal inflammation, systemic inflammation, and duration of menopausal years in postmenopausal women. Quintessence Int. 2018;49:123–31. 10.3290/j.qi.a39512 29234743

[117] Yakar N Türedi A Emingil G Şahin Ç Köse T Silbereisen A Bostanci N. Oral health and emotional well-being in premenopausal and postmenopausal women: a cross-sectional cohort study. BMC Women’s Health. 2021;211:338. 10.1186/s12905-021-01480-5 PMC8459505 34556103

[118] Agrawal R Ahmed H Soorgani N Naik L Reddy S Medabalmi M. Assessment of periodontal status in pre- and postmenopausal women with chronic periodontitis: a cross-sectional study. J Pharm Bioallied Sci. 2021;13:S997–9. 10.4103/jpbs.jpbs_145_21 35017915 PMC8686970

[119] Park KY Kim MH Choi SH Pang EK. Association of periodontitis with menopause and hormone replacement therapy: a hospital cohort study using a common data model. J Periodontal Implant Sci. 2023;53:184–93. 10.5051/jpis.2202480124 36468484 PMC10315258

[120] Nielsen TL Hagen C Wraae K Brixen K Petersen PH Haug E Visceral and subcutaneous adipose tissue assessed by magnetic resonance imaging in relation to circulating androgens, sex hormone-binding globulin, and luteinizing hormone in young men. J Clin Endocrinol Metab. 2007;92:2696–705. 10.1210/jc.2006-1847 17426100

[121] Sellau J Groneberg M Fehling H Thye T Hoenow S Marggraff C Androgens predispose males to monocyte-mediated immunopathology by inducing the expression of leukocyte recruitment factor CXCL1. Nat Commun. 2020;11:3459. 10.1038/s41467-020-17260-y 32651360 PMC7351718

[122] Araneo BA Dowell T Diegel M Daynes RA. Dihydrotestosterone exerts a depressive influence on the production of interleukin-4 (IL-4), IL-5, and gamma-interferon, but not IL-2 by activated murine T cells. Blood. 1991;78, 688–99. 10.1182/blood.V78.3.688.688 1830499

[123] Hughes TK Fulep E Juelich T Smith EM Stanton GJ. Modulation of immune responses by anabolic androgenic steroids. Int. J. Immunopharmacol. 1995;17: 857–63. 10.1016/0192-0561(95)00078-x 8788115

[124] Souza FR Rochitte CE Silva DC Sampaio B Passarelli M Santos MRD Fonseca GW Battaglia Filho AC Correa K do Val RM Yonamine M Pereira RMR Negrão CE Kalil-Filho R Alves MJNN. Coronary inflammation by computed tomography pericoronary fat attenuation and increased cytokines in young male anabolic androgenic steroid users. Arq Bras Cardiol. 2023;120:e20220822. 10.36660/abc.20220822 37991119 PMC10697680

[125] Tsatsanis C Elenkov A Leijonhufvud I Vaporidi K Tivesten Å Giwercman A. Sex hormone-dependent and -independent regulation of serum BAFF and TNF in cohorts of transgender and cisgender men and women. Endocr Connect. 2023;12:e220456. 10.1530/EC-22-0456 36607156 PMC9986405

[126] Santos JD Oliveira-Neto JT Barros PR Damasceno LEA Lautherbach N Assis AP Th17 cell-linked mechanisms mediate vascular dysfunction induced by testosterone in a mouse model of gender-affirming hormone therapy. Am J Physiol Heart Circ Physiol. 2022;323:H322–35. 10.1152/ajpheart.00182.2022 35714175

[127] Fujita T Kawata T Tokimasa C Tanne K. Influence of oestrogen and androgen on modelling of the mandibular condylar bone in ovariectomized and orchiectomized growing mice. Arch Oral Biol. 2001;46(1):57–65. 10.1016/s0003-9969(00)00094-7 11163596

